# Vitamin B12 Deficiency Is Prevalent Among Czech Vegans Who Do Not Use Vitamin B12 Supplements

**DOI:** 10.3390/nu11123019

**Published:** 2019-12-10

**Authors:** Eliška Selinger, Tilman Kühn, Magdalena Procházková, Michal Anděl, Jan Gojda

**Affiliations:** 1 Centre for Research on Diabetes, Metabolism and Nutrition of Third Faculty of Medicine, Charles University, Dept. of Medicine 2, University Hospital Kralovske Vinohrady, Prague 10000, Czech Republic; eliska.selingerova@lf3.cuni.cz (E.S.); magdalena.prochazkova@fnkv.cz (M.P.); michal.andel@lf3.cuni.cz (M.A.); 2 Division of Cancer Epidemiology, German Cancer Research Center (DKFZ), 69120 Heidelberg, Germany; t.kuehn@dkfz-heidelberg.de

**Keywords:** vegan, cobalamin deficiency, iron deficiency, anemia, alternative diet, plant-based diet

## Abstract

As not much is known about the prevalence and predictors of nutritional deficiencies among vegans in the Czech Republic, we evaluated whether supplement use and duration of adherence to the vegan diet are associated with the risk of cobalamin and iron deficiencies. Associations between self-reported supplementation and duration of vegan diet with biomarkers of cobalamin (serum cobalamin, holotranscobalamin, homocysteine, folate) and iron status (serum ferritin, iron binding capacity, transferrin and saturation of transferrin) were assessed by cross-sectional analyses of medical data from a clinical nutrition center. Data from 151 (72 females) adult vegans (age 18–67 years), who were free of major chronic diseases and 85 (40 females) healthy non-vegans (age 21–47 years) were analyzed. Overall, vegans had significantly lower cobalamin, hemoglobin and ferritin levels, but higher folate and MCV values compared to non-vegans. Vegans not using cobalamin supplements were at higher risk of low plasma cobalamin than regularly supplementing vegans (OR: 4.41, 95% CI 1.2–16.16 for cobalamin, OR: 19.18, 95% CI 1.02–359.42 for holotranscobalamin), whereas no significant differences in cobalamin status related to duration of the vegan diet were observed. Regularly supplementing vegans had similar levels of cobalamin/holotranscobalamin as non-vegans. Despite lower ferritin and hemoglobin levels, there was no indication of a higher risk of iron-deficiency among vegans. To conclude cobalamin deficiency risk depends on supplementation status and not on the duration of an exclusive vegan diet, which underlines the need to integrate cobalamin status monitoring and counselling on supplement use in routine clinical care in the Czech Republic.

## 1. Introduction

Veganism is defined as a philosophy and way of living, which seeks to exclude, as far as possible and practicable, all forms of exploitation of, and cruelty to, animals for food, clothing and any other purpose [1]. While a vegan diet is traditionally followed in different parts of India, veganism gained some attention as an alternative ethical movement in Western countries in the 1960s. Recent surveys indicate that the number of self-identified vegans in the USA increased from 0.4% in 2015 to 6% in 2017 [2]. In the Czech Republic, 1% of the population reported to be vegetarian in 2002, and about 2% in 2003 [3]. According to a current marketing survey performed in 2019, 1% of Czech population consider themselves as vegans (excluding all animal products) and 3% as vegetarians (excluding only meat). However, in younger adults (18–34 years) the proportion of vegetarians and vegans is 10% [4].

Based on the concept of the so-called rational vegan diet, promoted by vegan organizations and by some scientific societies, meat and meat products, dairy, eggs and honey can be replaced by legumes and grains as the main sources of protein and nutrients. A rational vegan diet should theoretically meet all of the requirements for nutrients except of cobalamin, which should be supplemented [5,6]. However, this approach may not be accepted by some members of the vegan community, and according to a systemic review from 2014 the prevalence rates of cobalamin deficiency in vegetarian and vegan adults and elderly range from 0% to 86.5%, with higher prevalence rates among vegans [7]. For example, 78% of Slovak vegans show laboratory signs of cobalamin deficiency based on the measurement of cobalamin levels in blood, according to one of the studies included in the review [8]. Beyond cobalamin supply, there is an ongoing discussion as to whether intakes of plant-derived iron, zinc, protein and total energy are sufficient among vegans, especially in areas such as post communistic Europe, where vegetarian and vegan diets do not have a long-term tradition as in Western European countries. At the same time, there is a lack of resources in the public health sector to address specific healthcare needs of vegans, arising from distinct nutrient patterns of the vegan diet in comparison to traditional western diet. As not much is known about the health status of vegans in the Czech Republic, we aimed to 1/assess cobalamin, and iron status and a risk of associated anemias among vegans and to 2/relate cobalamin deficiency to supplement use and duration of an exclusive vegan diet.

## 2. Materials and Methods

### 2.1. Study Population

Medical records of the out-patient department at a tertiary clinical nutrition center (University Hospital Kralovske Vinohrady, Prague, Czech Republic) were used for the present study. Data of vegan adults, who attended medical check-ups between 2012 and 2019, and volunteers from previous studies [9,10] of the department, who self-identified themselves as vegans (exclusive plant-based diet) were analyzed (n = 151). Non-vegan healthy volunteers, who were recruited for previous research projects of the department (Czech Rep. Ministry of Health grants 14,416 and 18-01-00040; Charles University grant 1,280,218), were used as controls subjects. Details on the duration of adherence to an exclusive vegan diet (self-reported, in years), supplementation habits (any supplementation containing any dose of cobalamin) were recorded, anthropometric measures were obtained, and health status was assessed. All study participants were free of major chronic diseases (i.e., diabetes, cancer, coronary heart disease, and stroke). The study was evaluated and approved by local ethical research committee of Third Faculty of Medicine, Charles University, Prague.

### 2.2. Laboratory Methods

Peripheral blood samples were obtained after an over-night fast. The biomarkers of interest were chosen and serum analyses were performed immediately in the ISO certified institutional laboratory by validated routine methods. For cobalamin and folate chemiluminescence immunoassay, for holotranscobalamin electrochemiluminiscent Elecsys active B12 assay and for iron FerroZine colorometry was used, all analyzed automatically on a Cobas 8000 system (Roche Diagnostics GmbH, Mannheim, Germany). Homocystein, ferritin (chemiluminescence immunoassay) and transferrin (immunoturbidimetry) using Advia Centaur XP (Siemens Healthcare Diagnostics, Tarrytown, NY, USA). Saturation of transferrin was calculated as TS = 3.98 × ([iron] μmol/L)/([transferrin] g/L) and total iron binding capacity was calculated as TIBC = [unbound iron capacity] μmol/L + [iron] μmol/L. As per hemoglobin (HGB) in whole blood, it was measured using photometry, hematocrit using direct impedance measurement and mean corpuscular volume MCV was calculated as hematocrit/red blood cell count (RBC). For cobalamin, holotranscobalamin, folate, ferritin and homocysteine reference values provided by manufacturer were used. Other reference intervals used are based on Czech population data [11].

### 2.3. Statistical Evaluation

For our statistical analyses we divided the study participants into groups based on the durations that they reported to have followed an exclusive vegan diet (non-vegans, <3 years: “short-term”; 3–7 years: “medium-term”; >7 years: “long-term”), as well as self-reported supplement use (non-vegans vs. “non-supplementing” vegans vs “irregularly supplementing” vegans vs. “supplementing” vegans). Kruskal-Wallis tests were carried to test for differences in blood biomarkers among vegans and non-vegans, and across the above-mentioned strata of supplement use and duration of the vegan diet. ANCOVA models were then used to evaluate differences in biomarkers between non-vegans and vegans stratified by the mentioned categories of supplement use and vegan diet duration adjusting for age, sex, and mutually adjusting for supplementation and duration, respectively. In addition, multivariable logistic regression models with the odds ratios (95% confidence intervals) of low plasma cobalamin levels (defined as cobalamin levels < 190 ng/L), increased homocysteine (defined as homocysteine levels > 15 µmol/L), decreased ferritin (ferritin < 30 µg/L) and other clinically relevant combinations of biomarkers were carried out to assess differences in biomarker levels by strata of cobalamin supplement use and duration of the vegan diet, again with mutual adjustment for these variables in addition to adjustment for age and sex. R (R: A language and environment for statistical computing) was used for all analyses and plots (R Core Team, Foundation for Statistical Computing, Vienna, Austria, 2018; URL, https://www.r-project.org/, using the tidyverse package (Hadley Wickham, 2017). Tidyverse: Easily Install and Load the ‘Tidyverse’. R package version 1.2.1. https://CRAN.R-project.org/package=tidyverse) and ggpubr package (Alboukadel Kassambara (2018) ggpubr: ‘ggplot2’ Based Publication Ready Plots. R package version 0.1.7 https://CRAN.R-project.org/package=ggpubr). A two-sided *p*-value of 0.05 was used to denote statistically significant associations.

## 3. Results

### 3.1. Characteristics of the Study Population

Characteristics of the study population are summarized in the Table 1. The average time vegan participants considered themselves to be vegan was 6.1 years (1.45–22.00 years). Overall, 40 vegan individuals were classified as “short-term” vegans (up to 3 years of adherence to the vegan diet, average 2.7), 67 as “medium-term” vegans (3–7 years, average 4.95), and 41 as “long-term” vegans (>7 years, average 11.3). With regard to supplement use, 17 vegans reported that they did not take any cobalamin supplements, while 92 vegans reported regular use of cobalamin supplements. Irregular cobalamin supplement use was reported by 39 vegan individuals.

### 3.2. Differences among Vegans and Non-Vegans in Selected Blood Markers

**Figure 1:**
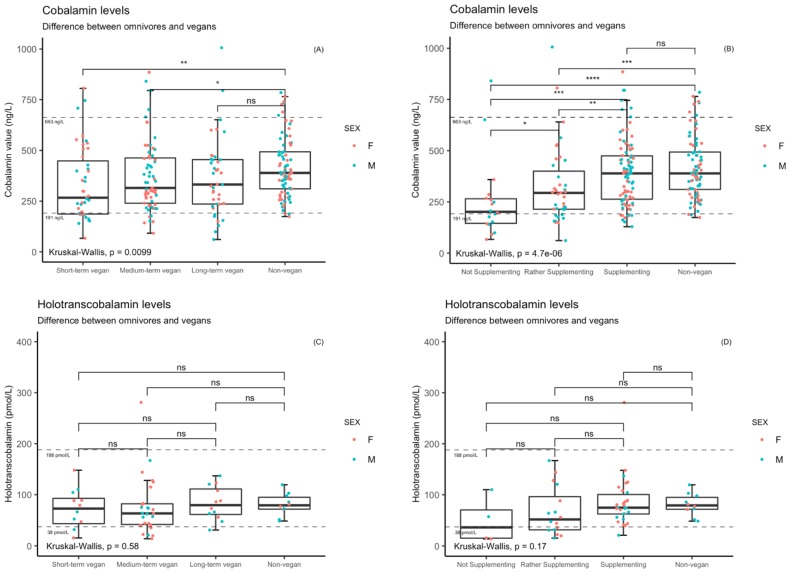
(**A**,**B**) Cobalamin levels stratified by the duration of vegan diet (compared to non-vegans); Cobalamin levels based on the supplementation habit (compared to non-vegans). Dashed lines indicated the reference interval of the marker. The coding of statistical significance: <0.0001 = ****, 0.0001–0.001 = ***, 0.001–0.01 = **, 0.01–0.05 = *, 0.05–1 = ns (non-significant). (**C**,**D**) Holotranscobalamin levels stratified by the duration of vegan diet (compared to non-vegans) and without showing the outlier values (2 male supplementing vegans holotranscobalamin = 682 and 521 pmol/L. The *p* values are calculated from all observations.) to visualize the major difference between groups; Holotranscobalamin levels based on the supplementation habit (compared to non-vegans)) and without the outlier values to visualize the major difference between groups. Dashed lines indicated the reference interval of the marker. The coding of statistical significance: <0.0001 = ****, 0.0001–0.001 = ***, 0.001–0.01 = **, 0.01–0.05 = *, 0.05–1, ns = non-significant.

**Figure 2:**
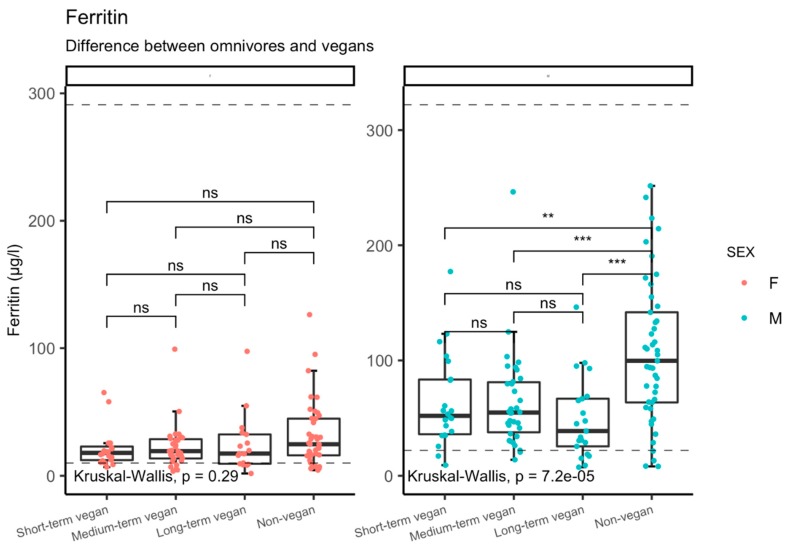
Ferritin levels, the stratification based on duration of vegan diet (compared to non-vegans) and based on sex. Dashed lines indicated the reference interval of the marker. The coding of statistical significance: 0.0001–0.001 = ***, 0.001–0.01 = **, 0.05–1 = ns (non-significant).

Results are summarized in Table 2 and Table 3. There were significant differences in the blood level of cobalamin, folate, hemoglobin, red blood cells’ MCV and ferritin across groups. No significant difference between vegans and non-vegans was found in the level of holotranscobalamin, homocysteine, with average value of 15 µmol/L in vegans and 14.1 µmol/L in non-vegans. The significant differences are also visualized in Figure 1 and Figure 2 in the text and Figures S1–S3 in the Supplementary Materials for the supplementation/duration subgroups, where relevant.

### 3.3. Cobalamin Status in Relation to Supplement Use and Duration of Vegan Diet

#### Differences in Markers Stratified by Supplementation Habit or Duration of Veganism

Differences among non-vegans and vegans’ subgroups across supplementation habit and/or duration of veganism are summarized in Table 4 and Table 5 and in Figure 1 and Figure 2 in the text and Figures S1–S3 in the Supplementary Materials.

Differences in vegan participants subgroups are described in detail in Supplementary Materials Section A. These differences in cobalamin concentrations depending on supplement use were statistically significant both in unadjusted Kruskal-Wallis tests and in linear regression models adjusted for age, sex. By contrast, no significant differences in cobalamin concentrations were observed between short-term, medium-term and long-term vegans. Overall, 23 out of 151 (15%) of vegans were classified as cobalamin deficient, defining deficiency as a cobalamin blood levels below 190 ng/L. Accordingly, logistic regression models adjusted for age, sex, and duration of adherence to the vegan diet showed a higher risk of lowered cobalamin levels as defined by cobalamin levels <190 ng/L among irregular supplement users 3.5 (95% CI 1.12–10.95) and non-users 7.69 (95% CI 2.11–28.05) compared to regular users. (see Supplementary Material, Figure S1–S3, Table S1–S2) Differences among non-vegans and vegans’ subgroups divided by supplementation habit and/or duration of veganism are summarized in Table 4 and Table 5. Multivariable logistic regression adjusted for age, sex and duration of veganism models revealed that non-supplementing vegans have higher risk of higher homocysteine levels (9.78, 95% CI 2.78–34.4) in comparison to regular supplementers (see Supplementary Material, Figure S1–S3, Table S1–S2).

Differences in holotranscobalamin levels between vegans and non-vegans were not significant in linear model adjusted for age, sex, supplementation and duration of veganism. The difference between vegans and non-vegans was also not significant according to Kruskal-Wallis or ANCOVA.

Among non-vegans, there were no people with holotranscobalamin levels below 37.5 pmol/L, among vegans it was the case in 15% of subjects (supplementing 5%, irregularly supplementing 32% and not supplementing 36.4%), despite the fact that there was no significant difference in levels of holotranscobalamin between vegans and non-vegans (and especially among regularly supplementing vegans and non-vegans).

### 3.4. Risk of Deficiency

#### 3.4.1. Cobalamin Metabolism

Vegans have significantly higher risk of cobalamin levels below 190 ng/L: OR 5.49 (95% CI 1.6–18.87). When defining deficiency based on the levels of cobalamin and homocysteine together, irregularly supplementing vegans have 3.89 (95% CI 1.13–13.45) higher and non-supplementing vegans have 21.48 (95% CI 5.49–84.02) higher risk for deficiency than regularly supplementing. Using combination of markers for assessment the risk of laboratory signs of cobalamin deficiency (cobalamin, homocysteine and folate values (cobalamin value < 100 ng/L; or cobalamin value between 100–200 ng/L and folate > 4.6 µg/L and homocysteine > 15 µmol/L), 16.9% of the vegan study population was cobalamin deficient; 5.56% among the supplement users, 17.9% among the irregular supplement users, and 52.9% among the non-users, while only 3 non-vegans out of 85 could be classified as cobalamin deficient. Compared to non-vegans, vegans have OR 7.05 (1.6–31.06) for having been classified as deficient.

With regard to duration of vegan diet, the highest prevalence of lowered cobalamin levels was found among short term vegans (28.2%) compared to mid-term (4.55%) and long-term (17.1%) while only 3.7% in non-vegans. In multivariable logistic regression analyses (adjusted for age, sex and supplementation habit), the lower prevalence of low levels of cobalamin among medium-term vegans in comparison to short-term vegans was significant. Of note, there were only two vegans with folate values below 4.6 µg/L (one male, short-term vegan and one medium-term vegan female, both in regularly supplementing group). For details in the differences among vegan subgroup see Supplementary Material, Section A).

Based on duration of vegan diet (compared to non-vegans), ORs were for short-term vegans: 15.07 (3.13–72.56), medium-term vegans: 1.96 (0.32–12.22) and for long-term vegans: 9.28 (1.76–48.9). Based on supplementation habits, there were significantly higher risks for not-supplementing and irregularly supplementing vegans: OR 18.89 (3.34–106.71) and 8.61 (1.81–49.93), respectively. The difference among supplementing vegans and non-vegans was not significant (OR 2.46, 0.51–11.89).

#### 3.4.2. Markers of Iron Deficiency

Anemia, defined as hemoglobin levels below 130 g/L for males or 120 g/L for females, was recorded in 12.87% of participants, 13.6% of the vegan participants (6.49% of the males, 21.4% of the females). In non-vegans, it was in 4.94% (10.8% or females, 0% for males). In the logistic regression model taking into account the age and sex the difference in the risk of lower hemoglobin among groups for vegans compared to non-vegans OR: 3.25, 95% CI 1.04–10.16). The lower values of MCV, defined as MCV < 82 fL, were found in 7.53% of vegans and 7.4% of non-vegans. In the logistic regression model, the difference was not significant (vegans compared to non-vegans OR: 1.05 (0.38–2.98). Ferritin values under 30 µg/L were found in 44.76% of study participants, 33.3% of non-vegans and 46.2% of vegans. In logistic regression, the difference in risk of the values of ferritin under 30 µg/L was for vegans (compared to non-vegans: OR 2.25, 95% CI 1.12–4.5). When looking on the combination of markers suggesting the ongoing iron deficiency, the combination of low hemoglobin and low ferritin values (HGB < 130/120 in males or females, respectively + ferritin < 30 µg/L), 7.41% of non-vegans and 11.6% of vegans could be classified a possibly deficient. The difference in risk was not significant in logistic regression model adjusting for age and sex: OR 1.71 (0.62–4.66). Similar results were obtained when looking on the combination of low ferritin, low iron and low iron binding capacity (ferritin < 30 µg/L OR iron < 7.2/6.6 (for males or females, respectively) µmol/L and iron binding capacity < 45 µmol/L), in which case 51% 4 of non-vegans and 50.1% of vegans had at least one pathological values, while the number was 40.5% for non-vegans, again the difference was not significant in logistic regression. For selected markers, more detailed analysis investigating the difference in the risk of values suggesting iron deficiency based on the duration of veganism in vegan participants was prepared (see Supplementary Material, Table S3).

## 4. Discussion

In the current study involving 151 Czech vegans and 85 non-vegan controls, we showed that vegans have significantly lower levels of cobalamin, hemoglobin and ferritin, but higher folate and MCV. The risk of laboratory signs of cobalamin deficiency among vegans was strongly related to the non-use of dietary supplements, and even irregular users of supplements were at higher risk for low levels of cobalamin and holotranscobalamin compared with regular users or non-vegans. By contrast, our study does not indicate that the duration of adherence to the vegan diet alone is related to the risk of cobalamin deficiency. Despite lower ferritin values among vegans (not related to the duration of vegan diet), the overall presence of the typical laboratory markers of iron deficiency anemia was low in both among vegans and non-vegans, and there were no statistically significant differences in the prevalence of such signs. Ferritin and hemoglobin values were significantly lower among vegan vs. non-vegan men, while no such difference was observed among women.

While evidence from the past years on homocysteine as a biomarker of cardiovascular disease risk is not consistent [12,13], our data do indicate that it may be one potential marker of suboptimal cobalamin supplementation among vegans. However, it should be noted that several vegan participants (n = 30) of our study, who were cobalamin sufficient according to their cobalamin levels (cobalamin > 190 ng/L), would have been classified as cobalamin deficient according to their homocysteine (homocysteine > 15 µmol/L, while folate > 4.6 µg/L). Despite the fact, that vegan study participants had a significantly higher risk of lower cobalamin levels overall compared to non-vegans, there was no such difference in homocysteine levels.

The observed tendency for higher MCV values among vegans was no longer statistically significant after adjustment for supplement use. Also, almost all of the participants in the study were in the normal range of MCV, despite having low cobalamin or showing a tendency for lower ferritin. Based on this finding, we assume that MCV may not be a valid marker for cobalamin or iron deficiency anemias in vegans, even though it is traditionally recommended for this purpose. Possibly, our observation can be either explained by higher levels of folate in vegans (masking the underlying cobalamin deficiency) or by lower ferritin levels (as a result of combination of cobalamin and borderline iron deficiency).

While the duration of adherence to the vegan diet was not associated with cobalamin, holotranscobalamin or homocysteine levels in our study, we observed a trend towards decreased iron load and storage with a longer vegan diet duration, ferritin being significantly lower in vegans than in non-vegans (not related to the duration of the vegan diet). Interestingly, many vegans in our study showed iron status parameters that clustered around the threshold value for iron deficiency, but only a minority of them had levels indicating iron deficiency. Although such borderline values could be interpreted as a risk factor for sideropenic anemia, low iron stores were also suggested to be partially responsible beneficial health effects of a vegan diet [14,15]. Higher iron load, as primarily reflected by higher ferritin values, is associated with cardiovascular disease and diabetes, and suspected to also be a risk factor for cancer development [16,17,18]. The interpretation of all other iron markers is complicated by their high intra-individual fluctuation, especially among females, that suggests that single examination may not be sufficient to assess iron status.

Given the increasing prevalence of alternative diets, guidelines for cobalamin deficiency assessment by blood markers according to which health professionals can be trained are needed. In this regard, it would have been of interest to evaluate other reliable markers of cobalamin deficiency used in practice, namely methylmalonic acid MMA [19,20] in our study population. Unfortunately, however, MMA has not been available in regular clinical practice in the Czech Republic. Though the absence of MMA limits our results, it of note that most general practitioners usually rely on the assessment of cobalamin only, often in combination with MCV, which may not reflect cobalamin deficiency in an accurate manner. Possibly, cobalamin assessments can serve as first line screening tool, with additional holotranscobalamin or MMA measurements in specialized laboratories in case of levels in the lower normal range.

Further limitations of the present study have to be considered. First, our vegan study population was a convenience sample of adults, who voluntarily attended healthcare checkups at the outpatient department of Clinical Nutrition. It is conceivable that this sample may have a higher degree of health consciousness together with greater trust in Western medicine and practicing physicians compared to other Czech vegans, who may be more prone to utilizing alternative medicine. Thus, it can be speculated that the overall prevalence of cobalamin deficiency among vegan adults in the Czech Republic may be higher than in the current sample of vegans. Our study did not include children, pregnant females, or lactating females, who may be particularly vulnerable to consequences of cobalamin deficiency. Indeed, borderline cobalamin status in pregnant women can be associated with increased risk of acquired newborn B12 deficiency and cobalamin alone may not be sufficient to diagnose deficiency among them, which is why MMA tests are required [21]. Future studies are needed to assess the prevalence and determinants of deficiencies in the mentioned vulnerable groups. As we relied on medical reports, only limited data was available concerning cobalamin supplementation, and we did not have information on the exact doses of supplemented cobalamin, limiting dose-response analyses. Of note, even in the group of long-term vegans without supplement use, there was one person reporting being vegan for 16 years with a cobalamin level 359 ng/L and a homocysteine level of 14 µmol/L. In addition, three other long-term vegans not using supplements had cobalamin values over 190 ng/L, suggesting, there could be an alternative source of cobalamin beyond classical supplements. However, this notion remains speculative, as supplement use was self-reported, and we were not able to further investigate other potential sources of cobalamin.

Our data may stimulate future research, and foster a more evidence-based debate about medical guidelines and recommendations targeting populations with alternative lifestyles in eastern European countries. There is clear indirect evidence that the popularity of alternative diets is increasing even in regions, in which the traditional cuisine is heavily based on animal products and where the availability of plant-based alternatives is not as high as in countries with a longer tradition of veganism. While we showed there is a substantial proportion of vegans, who do not supplement B12 sufficiently, the current shift in eating habits towards plant-based diets is not reflected by current public health recommendations and guidelines.

## 5. Conclusions

To conclude, we observed that Czech vegans are at higher risk of cobalamin/holotranscobalamin and ferritin deficiency overall. This difference was driven by irregular or non-use of cobalamin supplements in subgroups of the vegan study population, while regular supplement users were not at a higher risk of cobalamin deficiency. The duration of adherence to the vegan diet was not associated with risk for low cobalamin values. Public health services and healthcare professionals in the Czech Republic and other countries, where the vegan diet is becoming more popular, should meet the needs of vegans, especially regarding cobalamin status monitoring to prevent cobalamin deficiency.

## Figures and Tables

**Table 1:** Description of basic characteristics of vegans and omnivores.

Group	Age		BMI		Sex		Waist Circumference
	*n*	Mean Age	*n*	Mean BMI	Males	Females	Mean Waist Circumference
Vegans	151	32.8 ± 7.4	105	22.6 ± 2.7	78	73	81.5 ± 9.11
Omnivores	84	32.0 ± 7.0	84	23.7 ± 3.1	45	39	77.4 ± 9.98
*p*-value	0.394		0.005		0.779		0.002

*n* = number of patients with the value available for analysis, *p*-value: Kruskal-Wallis test.

**Table 2:** Mean values of blood markers related to cobalamin metabolism in vegans and non-vegans.

	Group	*n*	Mean	Min	Max	*p*-Value	ANCOVA *
Cobalamin (ng/L)	Vegans	148	359	61	1006	0.002	0.014
	Non-vegans	84	416	174	785		
HCY (µmol/L)	Vegans	147	14.7	5.8	65	0.607	0.440
	Non-vegans	84	14.1	7.2	29		
Folate (µg/L)	Vegans	146	13.0	3.63	40.5	<0.001	<0.001
	Non-vegans	84	8.74	2.53	21.0		
Holotranscobalamin (pmol/L)	Vegans	99	89.7	14.0	682	0.121	0.909
	Non-vegans	34	87.9	44.0	150		

*n* = number of patients with the value available for analysis, * ANCOVA: adjusted for SEX, AGE, reference values for the individual markers (reported accordingly to the clinical reference laboratory recommendations) are as follows: Cobalamin: 191–663 ng/L, Holotranscobalamin: 37.5–188 pmol/L, Homocysteine: 3–17 µmol/L, Folate: 4.6–18.7 µg/L.

**Table 3:** Mean values of blood markers related to iron metabolism in vegans and non-vegans.

	Group	*n*	Mean	Min	Max	*p*-Value	ANCOVA
Ferritin (µg/L)	Vegans	145	43.6	1.8	246	0.001	<0.001
	Non-vegans	81	72.2	4.4	252		
Iron (µmol/L)	Vegans	150	19.0	3.3	65.6	0.392	0.925
	Non-vegans	84	19.2	5.2	41.5		
Iron Binding Capacity (µmol/L)	Vegans	150	56.8	38	88	0.327	0.326
	Non-vegans	84	56.3	37	92		
Transferrin (g/L)	Vegans	139	2.74	1.74	4.18	0.396	0.993
	Non-vegans	84	2.74	1.92	4.52		
Saturated Transferrin (%)	Vegans	135	28.7	3.4	79.1	0.614	0.930
	Non-vegans	68	28.8	7.5	70.9		
Hemoglobin (g/L)	Vegans	150	138	97	169	0.022	<0.001
	Non-vegans	84	142	111	169		
MCV (fL)	Vegans	149	88.5	87.2	103	0.009	0.022
	Non-vegans	84	87.2	74.3	96.4		

*n* = number of patients with the value available for analysis, *p*-value for Kruskal-Wallis, ANCOVA adjusted for SEX, AGE; reference values for the individual markers (reported accordingly to the clinical reference laboratory recommendations) are as follows: MCV, mean corpuscular volume 82–92 fL; hemoglobin: F: 120–160 g/L, M:135–175 g/L; Iron: F: 6.6–28.0 µmol/lM: 7.2–29.0 µmol/L; Iron binding capacity: 45–72 µmol/L; ferritin: F:10–291 µg/L M: 22–322 µg/L; transferrin: 2–3.6 g/L; saturation of transferrin: 16–45%.

**Table 4:** Mean values of selected blood markers in groups across vegan cobalamin supplement use as compared to non-vegans.

	Supplement Use	*n*	Mean	Min	Max	*p*-Value	ANCOVA
Cobalamin (ng/L)	Regular	90	398	129	885	<0.001	0.002
	Irregular	39	329	61	1006		
	None	17	256	67	841		
	Non-vegan	84	389	174	785		
Holotranscobalamin (pmol/L)	Regular	63	104	21.1	682	<0.001	0.06
	Irregular	25	72.2	15.5	167		
	None	11	46.1	14	110		
	Non-vegans	34	87.9	44.0	150		
Homocysteine (µmol/L)	Regular	90	13.5	5.8	33.8	0.001	<0.001
	Irregular	37	15.2	7.9	65		
	None	17	20.3	9.8	46.4		
	Non-vegans	84	14.1	7.2	29		
MCV (fL)	Regular	91	88.4	79	98.3	0.04	0.63
	Irregular	38	88.6	63.9	103		
	None	17	89.5	84.6	94.8		
	Non-vegans	84	87.2	74.3	96.4		

*n* = number of patients with the value available for analysis, *p*-value for Kruskal-Wallis, ANCOVA adjusted for SEX, AGE, DURATION OF VEGAN DIET; reference values for the individual markers (reported accordingly to the clinical reference laboratory recommendations) are as follows: Cobalamin: 191–663 ng/L; Holotranscobalamin: 37.5–188 pmol/L, Homocysteine: 3–17 µmol/L; Folate: 4.6–18.7 µg/L; MCV, mean corpuscular volume 82–92 fL.

**Table 5:** Mean values of selected blood markers in groups divided by the duration of the veganism and compared to non-vegans.

	Duration of Veganism	*n*	Mean	Min	Max	*p*-Value	* ANCOVA
Cobalamin (ng/L)	Longterm vegan	41	341	61	795	0.02	0.05
	Medium-term vegan	66	388	92	1006		
	Short-term vegan	39	344	67	806		
	Non-vegan	84	389	174	785		
Holotranscobalamin (pmol/L)	Long-term vegan	27	103	31.1	521	0.15	0.58
	Medium-term vegan	53	88.2	14.0	682		
	Short-term vegan	19	74.5	15.5	148		
	Non-vegan	34	87.9	44.0	150		
Homocysteine (µmol/L)	Long-term vegan	39	16.9	6.2	65	0.332	0.028
	Medium-term vegan	65	13.3	6.8	28.9		
	Short-term vegan	40	14.9	5.8	46.4		
	Non-vegans	84	14.1	7.2	29		
Folate (µg/L)	Long-term vegan	40	12.9	4.98	25.0	<0.001	<0.001
	Medium-term vegan	65	13.0	3.63	40.5		
	Short-term vegan	37	13.0	4.5	21.9		
	Non-vegan	24	9.75	3.64	20.0		
MCV (fL)	Long-term vegan	40	87.8	63.9	103	0.03	0.06
	Medium-term vegan	66	88.8	79	96.1		
	Short-term vegan	40	88.8	80.4	98.3		
	Non-vegans	84	87.2	74.3	96.4		
Ferritin (µg/L)	Long-term vegan	38	39.4	1.8	146	0.005	<0.001
	Medium-term vegan	65	41.6	3.6	246		
	Short-term vegan	39	51.1	6.8	177		
	Non-vegans	81	72.2	4.4	252		

*n* = number of patients with the value available for analysis, * ANCOVA: adjusted for SEX, AGE, DURATION OF VEGAN DIET, reference values for the individual markers (reported accordingly to the clinical reference laboratory recommendations) are as follows: Cobalamin: 191–663 ng/L, Holotranscobalamin: 37.5–188 pmol/L, Homocysteine: 3–17 µmol/L, Folate: 4.6–18.7 µg/L MCV, mean corpuscular volume 82–92 fL.

## Supplementary Materials

The following are available online at https://www.mdpi.com/2072-6643/11/12/3019/s1, Figure S1: Folate levels between vegans and non-vegans, Figure S2: Difference in HGB between vegans and non-vegans, division into subgroups based on duration of vegan diet and sex, Figure S3: (**a**) MCV based on the duration of veganism (compared to non-vegans); (**b**) MCV based on the supplementation habit (compared to non-vegans), Table S1: The risk of values suggesting cobalamin deficiencies in relation to supplement use (with adjustment for age, sex and the duration of vegan diet), Table S2: The risk of values suggesting nutritional deficiencies in relation to duration of being vegan, Table S3: The risk of values suggesting iron deficiency in relation to duration of being vegan (with adjustment for age, sex and supplementation habit).
