# Supplementary material for: Vitamin B12 Deficiency Is Prevalent Among Czech Vegans Who Do Not Use Vitamin B12 Supplements
Source: Nutrients. 2019 Dec 10;11(12):3019. doi: 10.3390/nu11123019 (PMC6950550; doi:10.3390/nu11123019)
Supplement: Supplementary file 1 [file nutrients-11-03019-s001.pdf]

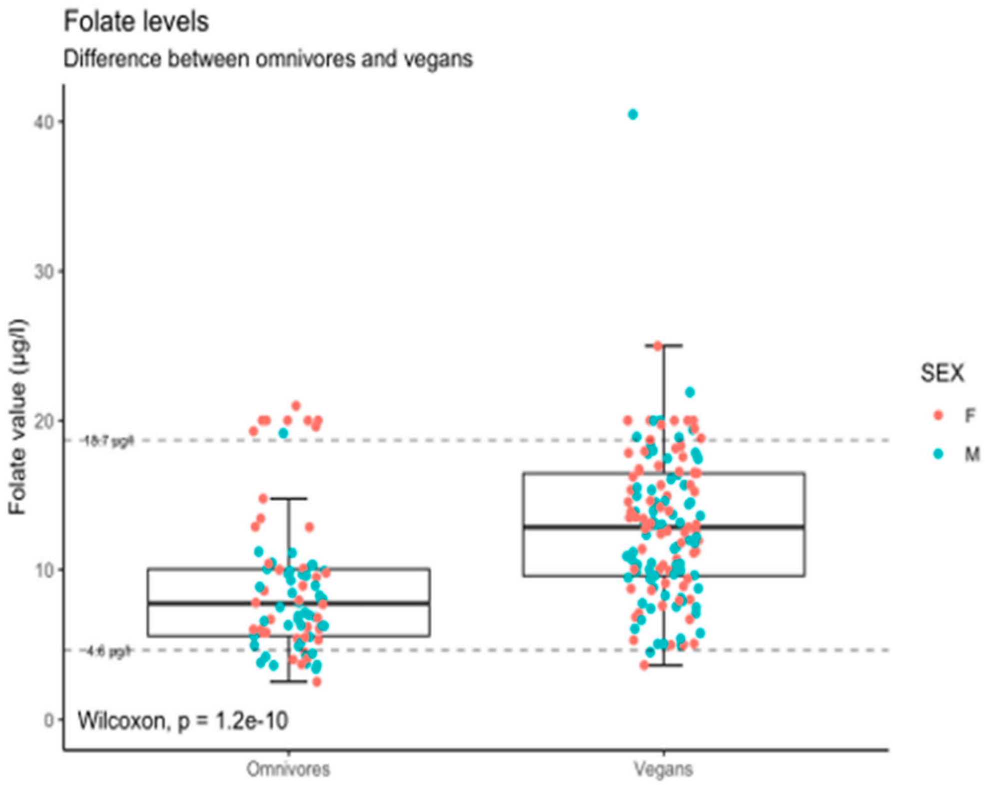

Supplementary Figure S1. Folate levels between vegans and non-vegans; Dashed lines indicated the reference interval of the marker. The coding of statistical significance: < 0.0001 = ****, 0.0001 – 0.001 = ***, 0.001 – 0.01 = **, 0.01-0.05 = *,0.05 – 1 = ns.

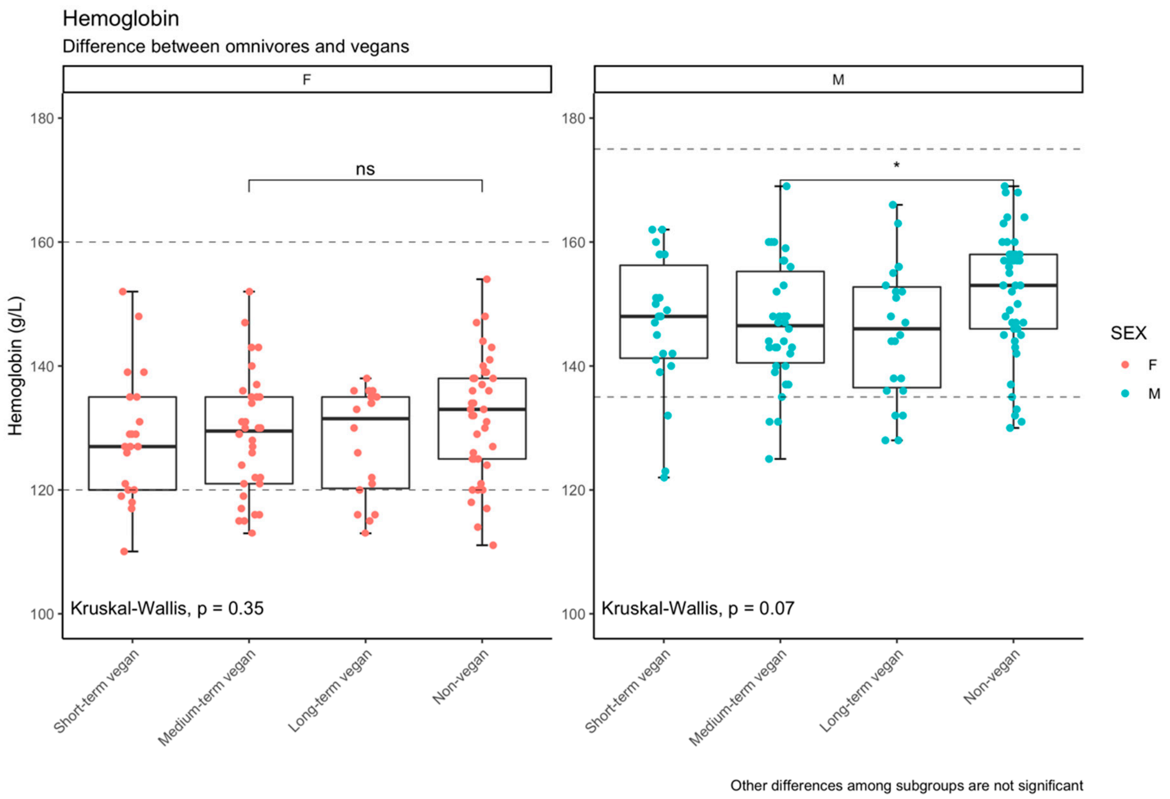

Supplementary Figure S2. Difference in HGB between vegans and non-vegans, division into subgroups based on duration of vegan diet and sex. Dashed lines indicated the reference interval of the marker. The coding of statistical significance: < 0.0001 = ****, 0.0001 – 0.001 = ***, 0.001 – 0.01 = **, 0.01-0.05 = *,0.05 – 1 = ns.

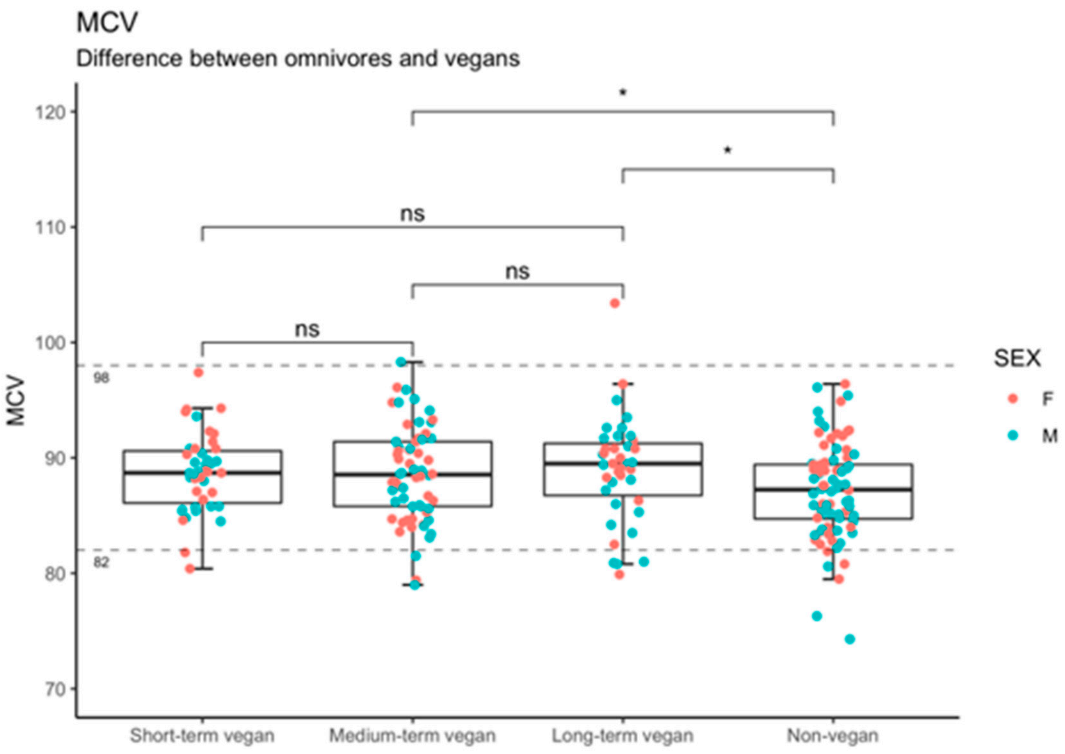

(**a**)

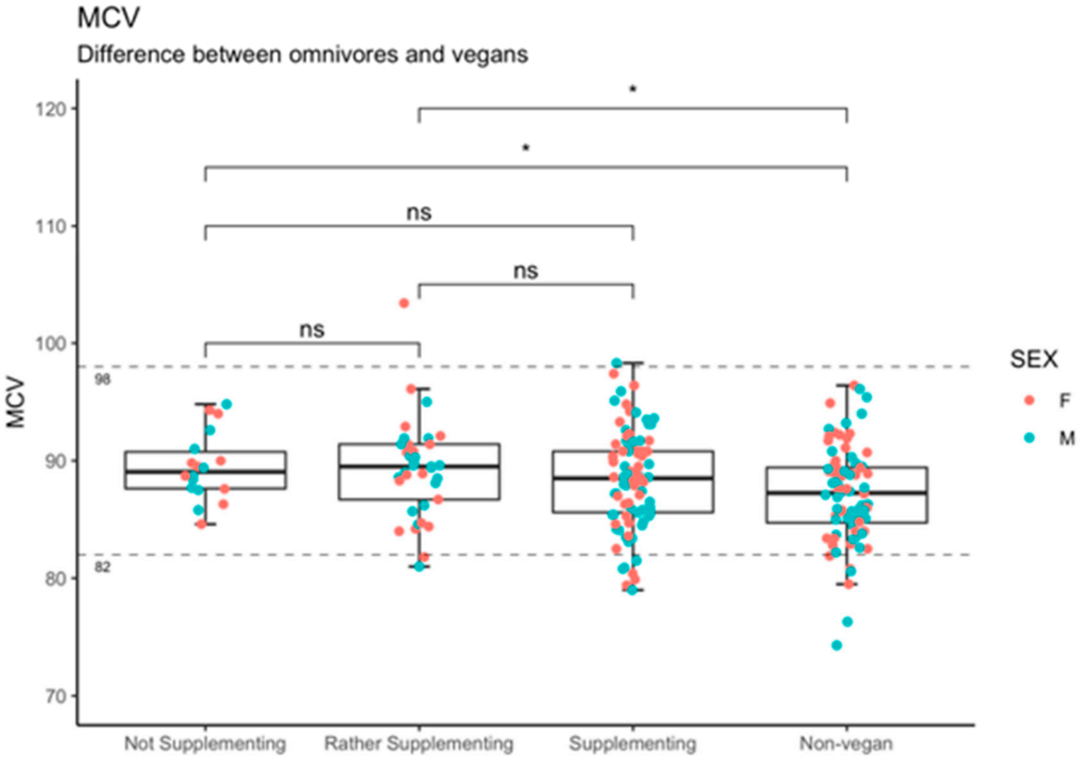

(**b**)

Supplementary Figure S3. (**a**) MCV based on the duration of veganism (compared to non-vegans); (**b**) MCV based on the supplementation habit (compared to non-vegans). Dashed lines indicate the reference interval of the marker. The coding of statistical significance: < 0.0001 = ****, 0.0001 – 0.001 = ***, 0.001 – 0.01 = **, 0.01-0.05 = *,0.05 – 1 = ns.

Supplementary table S1. The risk of values suggesting cobalamin deficiencies in relation to supplement use (with adjustment for age, sex and the duration of vegan diet).

	n	% def.	supplement use	*n*	%def.	OR	CI 95%	criteria
Cobalamin	146	15.75	Regular	90	8.89	ref.	-	cobalamin < 190 ng/l
			Irregular	39	20.5	3.50	1.12- 10.95	
			None	17	41.2	7.69	2.11 – 28.05	
Holotranscobalamin	100	15	Regular	63	4.76	Ref.	_	cobalamin < 37.5 pmol/L
			Irregular	25	33.3	28.12	2.57 – 307.0	
			None	12	32.0	19.18	1.02 – 359.0	
Homocysteine	144	36.81	Regular	90	27.8	ref.	-	Homocysteine >= 15 μmol/l
			Irregular	37	40.5	2.02	0.86 - 4.76	
			None	17	76.5	9.60	2.69 - 34.20	
Vitamin B12 combined	146	14,4	Regular	90	5.56	ref.	-	cobalamin < 100 ng/l or cobalamin < 200 ng/l with folate >= 4.6 μg/l and homocysteine > 15 μmol/l
			Irregular	39	17.9	5.06	1.35 – 18.96	
			None	17	52.9	29.47	6.38 - 135.99	
MCV increased	146	17.12	Regular	91	17.6	ref.	-	MCV > 92 fl
			Irregular	38	13.2	0.67	0.22- 2.02	
			None	17	23.5	1.48	0.47 – 4.09	

n = number of patients with the value available for analysis, % def. = percentage of patients with suspected deficit based on this value, OR = odds ratio, CI 95% = 95% confidence interval.

Supplementary table S2. The risk of values suggesting nutritional deficiencies in relation to duration of being vegan.

The risk of values suggesting cobalamin deficiencies in relation to duration of being vegan (with adjustment for age, sex and supplementation habit)								
	n	% path.	**duration**	**n**	**%**	**OR**	**CI 95%**	criteria
Cobalamin	146	15.75	Long-term	41	19.5	0.58	0.18 - 1.89	cobalamin < 190 ng/l
			Medium-term	66	6.06	0.16	0.04 - 0.58	
			Short-term	39	28.2	ref.	-	
Homocysteine	144	36.81	Long-term	39	43.6	0.96	0.35 - 2.67	Homocysteine >= 15 µmol/l
			Medium-term	65	26.2	0.43	0.17 - 1.05	
			Short-term	40	47.5	ref.	-	
Vitamin B12 combined	146	14,4	Long-term	41	17.1	0.60	0.16 - 2.25	cobalamin < 100 ng/l or cobalamin >= 100 ng/l but < 200 ng/l with folate >= 4.6 µg/l and homocysteine > 15 µmol/l
			Medium-term	66	4.55	0.08	0.02 - 0.41	
			Short-term	39	28.2	ref.	-	
MCV increased	146	17.12	Long-term	40	15	0.94	0.25 - 3.48	MCV > 92 fl
			Medium-term	66	19.7	1.39	0.47 – 4.09	
			Short-term	40	15	ref.	-	

n = number of patients with the value available for analysis, % def. = percentage of patients with suspected deficit based on this value, OR = odds ratio, CI 95% = 95% confidence interval.

Suplementary Table S3: The risk of values suggesting iron deficiency in relation to duration of being vegan (with adjustment for age, sex and supplementation habit).

	n	% path.	**duration**	**n**	**%**	**OR**	**CI 95%**	**criteria**
Decreased HGB	146	15.75	Long-term	41	17.1	1.26	0.32 – 4.94	M: HGB < 130 g/L<br>F: HGB < 120g/L
			Medium-term	66	12.1	0.78	0.23 - 2.69	
			Short-term	40	12.5	ref.	-	
MCV decreased	145	7.53	Long-term	40	15	14.62	1.67 – 127.55	MCV < 82 fl
			Medium-term	66	4.55	1.33	0.18 - 9.63	
			Short-term	40	5	ref.	-	
Ferritin	142	14,4	Long-term	38	47.4	1.14	0.34 - 3.81	Ferritin < 30 μg/L
			Medium-term	65	49.2	0.91	0.33 - 2.53	
			Short-term	39	41.0	ref.	-	
Decreased HGB, decreased ferritin	146	11.64	Long-term	40	17.5	2.07	0.46 - 9.12	M: HGB < 130 g/L<br>F: HGB < 120g/L<br>and ferritin < 30 μg/L
			Medium-term	66	9.09	0.69	0.17 – 2.76	
			Short-term	40	10.0	ref.	-	
Decreased ferritin OR decreased iron OR decreased iron binding capacity	141	51.77	Long-term	38	50.0	1.14	0.34 – 3.81	ferritin < 30 OR μg/L OR iron < M 7.2/ F 6.6 μmol/L OR iron binding capacity < 45 μmol/L
			Medium-term	65	55.4	0.91	0.33 - 2.53	
			Short-term	38	47.4	ref.	-	

n = number of patients with the value available for analysis, % def. = percentage of patients with suspected deficit based on this value, OR = odds ratio, CI 95% = 95% confidence interval.
